# Identification of DNA methylation-driven genes and construction of a nomogram to predict overall survival in pancreatic cancer

**DOI:** 10.1186/s12864-021-08097-w

**Published:** 2021-11-03

**Authors:** G.C Deng, D.C Sun, Q Zhou, Y Lv, H Yan, Q.L Han, G.H Dai

**Affiliations:** 1 Senior Department of Oncology, The Fifth Medical Center of Chinese People’s Liberation Army (PLA)General Hospital, Beijing, China; 2 School of Medicine, Nankai University, Tianjin, China

**Keywords:** DNA methylation, Nomogram, Risk score, Prognosis, Pancreatic cancer

## Abstract

**Background:**

The incidence and mortality of pancreatic cancer (PC) has gradually increased. The aim of this study was to identify survival-related DNA methylation (DNAm)-driven genes and establish a nomogram to predict outcomes in patients with PC.

**Methods:**

The gene expression, DNA methylation database, and PC clinical samples were downloaded from TCGA. DNAm-driven genes were identified by integrating analyses of gene expression and DNA methylation data. Survival-related DNAm-driven genes were screened via univariate, least absolute shrinkage and selection operator (LASSO), and multivariate Cox regression analyses to develop a risk score model for prognosis. Based on analyses of clinical parameters and risk score, a nomogram was built and validated. The independent cohort from GEO database were used for external validation.

**Results:**

A total of 16 differentially expressed methylation-driven genes were identified. Based on LASSO Cox regression and multivariate Cox regression analysis, six genes (FERMT1, LIPH, LAMA3, PPP1R14D, NQO1, VSIG2) were chosen to develop the risk score model. In the Kaplan–Meier analysis, age, T stage, N stage, AJCC stage, radiation therapy history, tumor size, surgery type performed, pathological type, chemotherapy history, and risk score were potential prognostic factors in PC (*P* < 0.1). In the multivariate analysis, stage, chemotherapy, and risk score were significantly correlated to overall survival (*P* < 0.05). The nomogram was constructed with the three variables (stage, chemotherapy, and risk score) for predicting the 1-year, 2-year, and 3-year survival rates of PC patients. Nomogram performance was assessed by receiver operating characteristic (ROC) curves and calibration curves. 1-year, 2-year and 3-year AUC of nomogram model was 0.899, 0.765 and 0.776, respectively.

**Conclusions:**

In our study, we successfully identified the six DNAm-driven genes (FERMT1, LIPH, LAMA3, PPP1R14D, NQO1, VSIG2) with a relationship to the outcomes of PC patients. The nomogram including stage, chemotherapy, and risk score could be used to predict survival in PC patients.

**Supplementary Information:**

The online version contains supplementary material available at 10.1186/s12864-021-08097-w.

## Introduction

Pancreatic cancer (PC), a malignant tumor with uniformly poor outcomes, is the sixth leading cause of cancer-related mortalities in China [1]. PC remains highly lethal, with a 5-year survival less than 9 % [2]. Currently, surgical treatment and systemic chemotherapy are the preferred therapeutic approaches for PC. Monotherapy (such as gemcitabine, S-1, capecitabine) is suitable for patients with poor performance [3]. Combination regimens, such as gemcitabine-based combinations or FOLFIRINOX (irinotecan, oxaliplatin, 5-FU/leucovorin), are appropriate for patients with good performance [4–7]. The median survival time of advanced PC patients is less than 12 months despite clinical therapeutic research development [8]. In addition, patients with PC are usually diagnosed in the advanced stages due to limitations in early diagnosis [9]. In PC, CA 19-9 is the most widely used tumor marker for early diagnosis, predicting survival, and monitoring therapeutic efficacy. However, the diagnostic value of CA 19-9 is limited due to its 70–80 % sensitivity and 80–90 % specificity [10]. Therefore, exploration of effective biomarkers for improving early diagnosis and prognosis is very important.

With the development of molecular biology technologies, increasing attention has been paid to efficient gene prediction. An increasing number of studies have attempted to identify prognostic genes at the mRNA level and have constructed different gene prognostic models to improve prognosis [11, 12]. For example, Meng-wei et al. identified a nine-gene prognostic model (MET, KLK10, COL17A1, CEP55, ANKRD22, ITGB6, ARNTL2, MCOLN3, and SLC25A45) with effective predictive ability [13].

Gene expression levels can be influenced by epigenetic dysregulation [14]. DNA methylation is a pivotal element for epigenetic modification and plays an important role in regulating genes and maintaining genome stability [15]. Aberrant DNA methylation of CpG islands in the promoter, which regulates the expression of tumor-related genes, is involved in carcinogenesis [16]. Previous studies have shown that hypermethylation of antioncogenes or hypomethylation of oncogenes can lead to tumorigenesis [17]. DNA methylation is also an important biomarker, which may be used for clinical diagnosis and prognosis in different tumor types [18, 19]. Jun-yu et al. demonstrated that DNA methylation-driven gene (SPP1 and LCAT) models exhibited good performance in the diagnosis and estimation of prognosis in hepatocellular cancer [20]. Yi et al. validated a survival prognostic model based on DNA methylation-driven genes (PODN, NPY, MICU3, TUBB6, RHOJ, MYO1A) and showed that it had good predictive ability in gastric cancer [21].

In this study, we screened effective PC-related DNA methylation-driven genes by merging methylation and mRNA expression profiles from TCGA (The Cancer Genome Atlas) database. We constructed a model based on DNA methylation-driven genes to predict outcomes in PC.

## Materials and methods

### Data preparation

**Fig. 1:**
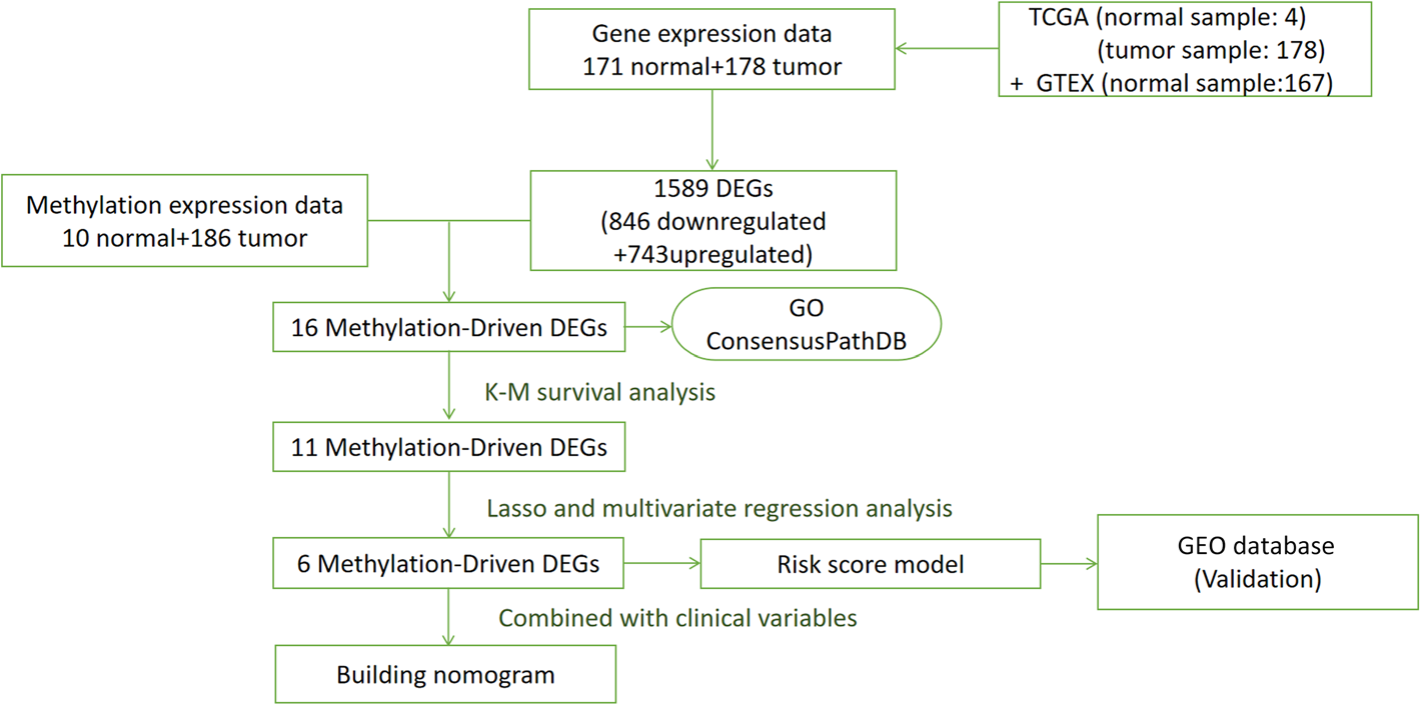
Flow chart of the identification and exploration methylation-driven genes in pancreatic cancer

We downloaded clinical survival data and DNA methylation of PC from TCGA dataset (https://portal.gdc.cancer.gov/). The mRNA expression of TCGA and GTEx (Genotype-Tissue Expression) was downloaded from the UCSC Xena website (https://xenabrowser.net/datapages/). There were 195 samples with DNA methylation data (10 normal and 185 tumor), 182 samples with mRNA expression data (4 normal and 178 tumor), and 185 cases with clinical data from TCGA. Additionally, 167 data points for normal mRNA expression were obtained from GTEx. The study flowchart is shown in Fig. 1.

### Identification of differentially expressed genes (DEGs)

First, we merged the mRNA expression data from TCGA (4 normal and 178 tumor mRNA expression) and GTEx (167 normal mRNA expression) with the Limma package in R. Then, we identified the DEGs (differentially expressed genes between normal and tumor tissues, including upregulated and downregulated genes) from the merged data (171 normal and 178 tumor mRNA expression) with the Limma package in R. Cutoff criteria were | Log2FC | > 2, and false discovery rate (FDR) < 0.05.

### Screening for DNA methylation (DNAm)-driven genes

We performed a comprehensive analysis to acquire three data matrices (gene expression data, normal DNA methylation, and tumor DNA methylation data). Then, we used the MethylMix package in R to screen the DNAm-driven genes. First, MethylMix was used to compare the methylation state of tumor tissues with that of normal tissues. Correlation analyses were performed between the gene expression data of DEGs and DNAm data to distinguish the DNA methylation events, which may affect gene expression. Second, a mixture model of gene methylation state was built. Third, the differential DNA methylation state between tumor and normal tissues was calculated via Wilcoxon rank sum test. *P* < 0.05, and Cor < − 0.3 were set as the cutoff criteria.

### Functional enrichment analysis and pathway analysis

Gene ontology (GO) analysis was performed using the clusterProfiler package in R. GO analysis contained cellular component, biological process, and molecular function. We applied the GOplot package in R to visualize the data. Pathway analysis of the methylation-driven genes was conducted with ConsensusPathDB (http://cpdb.molgen.mpg.de/). *P* < 0.05 was the cutoff criterion.

### Risk score model construction

First, we utilized univariate Cox regression to screen for survival related DNAm-driven genes. Second, we applied Cox LASSO regression to further narrow the range of the candidate DNAm-driven genes using the glmnet package in R. LASSO regression is a method that shrinks regression coefficients toward zero by utilizing an L1 penalty[22]. This method can also decrease dimension and avoid collinearity between the variables. Third, we used multivariate Cox regression to further select the genes associated with survival. The target gene risk score is equal to the multivariate Cox regression coefficient (β) multiplied by its mRNA level. Then, X-tile was applied to stratify patients into low- and high-risk groups using the optimal cutoff value. The performance of the risk model was validated by ROC curve with the survivalROC package in R.

### Validation of the risk score model

The potential predictive value of the risk score model was validated in the GSE21501, GSE57495, GSE78229 and GSE62452 cohort. They were downloaded from the Gene Expression Omnibus (GEO) (https://www.ncbi.nlm.nih. gov/geo/). We combined GSE21501 and GSE57495 datasets into one data set for external validation.

### Screening of clinical data and clinical variables

There were 185 clinical cases in TCGA database. We deleted the cases with a follow-up time ≤30 days and incomplete clinical information. Ultimately, 91 cases were selected to perform further survival analyses. Different clinical variables were utilized including age, gender, grade, T stage, N stage, M stage, AJCC stage, alcohol history, pancreatitis history, diabetes history, lymph node counts, tumor size, surgery type performed, pathological type, primary tumor location, radiation therapy history, and chemotherapy history.

### Development and validation of the nomogram

To select potential risk factors, the associations of each clinical variable with overall survival (OS) were estimated using the Kaplan–Meier method. The variable with a P value < 0.1 was selected for further analysis. In the Cox proportional hazards regression model, we used backward stepwise selection with AIC (Akaike information criterion) to identify the final prognostic variable. The variable with a statistical significance level of 0.05 was added to the nomogram model. The nomogram was used to predict the 1-year, 2-year, and 3-year OS rates with the rms package in R.

The performance of the model was estimated using the C statistic and calibration. The C statistic was used to evaluate the discriminating ability of the model and is equal to the area under the receiver operating characteristic (ROC) curve. Calibration estimated the accuracy of the model and was visualized by calibration plot. The nomogram was validated by the bootstrap method with 1,000 resamples. We also applied the ROC curve to measure the accuracy of the nomogram.

### Statistical analysis

All statistical analyses were performed using R (version 4.0.0, http://www.r-project.org/), and X-tile version 3.6.1 (Yale University, CT, USA) was used to find the optimal cut-off value for stratifying patients[23]. *P* < 0.05 was considered statistically significant. All methods in our manuscript were performed in accordance with the relevant guidelines and regulations.

## Results

### Identification of DEGs in PC

The mRNA expression between 178 PC tissues and 171 normal tissues was compared and 1,589 DEGs (|Log_2_FC| > 2, FDR < 0.05) were used for further study. Among these DEGs, 743 were upregulated and 846 were downregulated (Table S1, Figure S1).

### Identification of DNAm-driven genes in PC

**Fig. 2:**
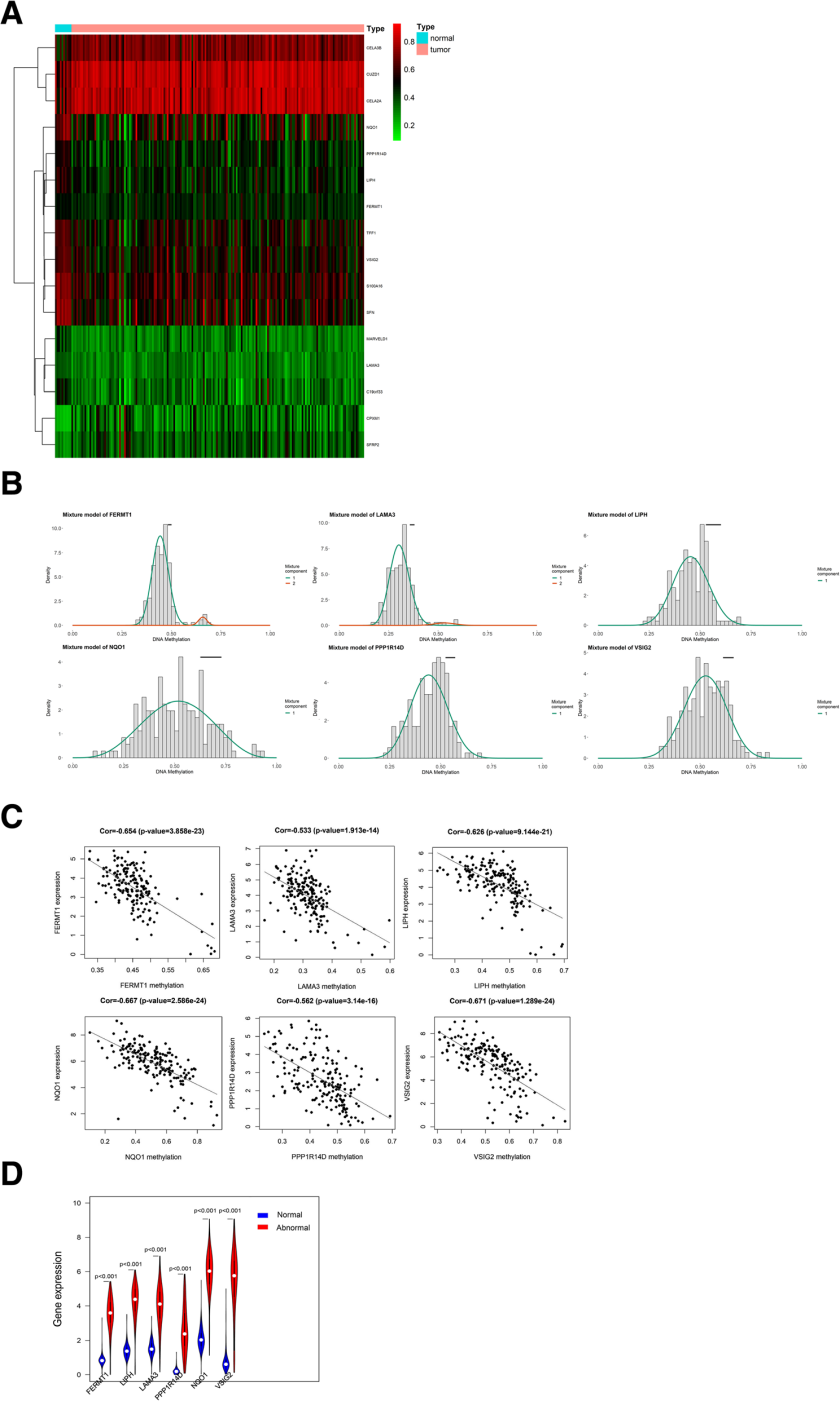
Identification of DNAm-driven genes. **A** Heatmap of 16 DNAm-driven genes in PC. **B** Mixture model of the six methylation-driven genes. The horizontal axis indicates the degree of methylation and the vertical of axis indicates the distribution of methylation in tumor samples. The black bar represents the methylation level in normal samples. **C** Correlation analysis between the mRNA expression level and DNA methylation level of the six DNAm-driven genes. The x-axis and y-axis indicate the DNA methylation level and mRNA expression level, respectively. **D** Violin plot. mRNA level of the six DNAm-driven genes

We applied the MethylMix analysis to screen the DNAm-driven genes. A total of sixteen DNAm-driven genes (eleven hypermethylated genes and five hypomethylated genes) were screened. An adjusted *P* value less than 0.05 between hypermethylated and hypomethylated groups and a correlation coefficient less than -0.3 between gene expression and DNA methylation were set as criteria for screening methylation-driven genes (Table S2). The methylation expression levels of sixteen DNAm-driven genes are shown in the heatmap (Fig. 2 A). Among these DNAm-driven genes, the methylation expression levels of six genes are shown in Fig. 2 B, C, D.

### Functional enrichment and pathway analysis of DNAm-driven genes

**Fig. 3:**
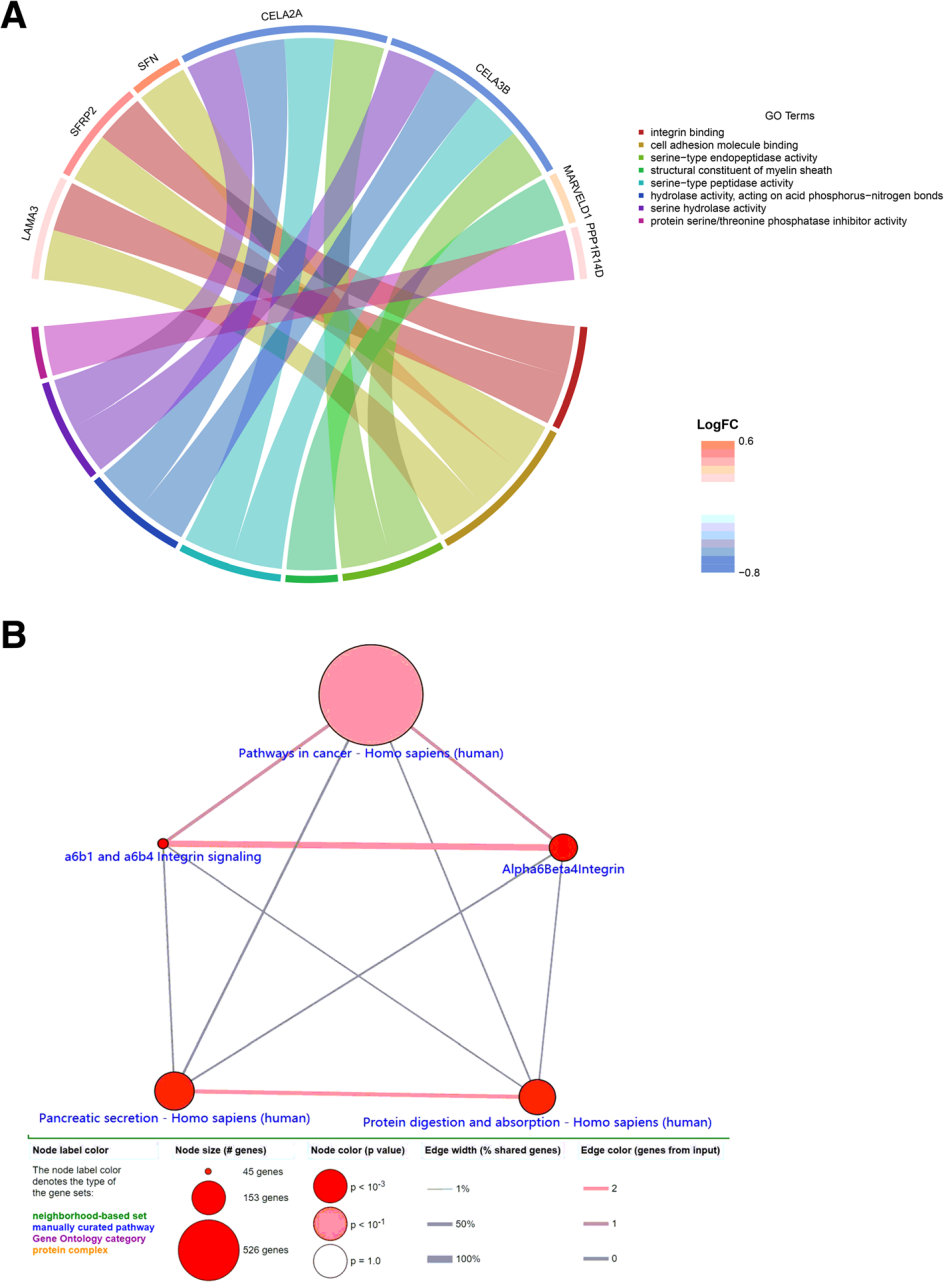
**A** GO analysis of sixteen DNAm-driven genes. **B** Pathway analysis of 16 DNAm-driven genes

Analysis of the function of DNAm-driven genes in PC was conducted by GO enrichment analysis with the clusterProfiler package in R. Functional enrichment analysis showed that DNAm-driven genes were enriched in molecular function (MF) such as cell adhesion molecule binding, integrin binding, and serine−type endopeptidase activity (Fig. 3 A). Pathway enrichment analysis showed that DNAm-driven genes were enriched in pancreatic secretion, protein digestion, and absorption, Alpha6Beta4Integrin and a6b1 and integrin signaling (*P* < 0.001) (Fig. 3 B).

### Development of the risk score model for PC

**Fig. 4:**
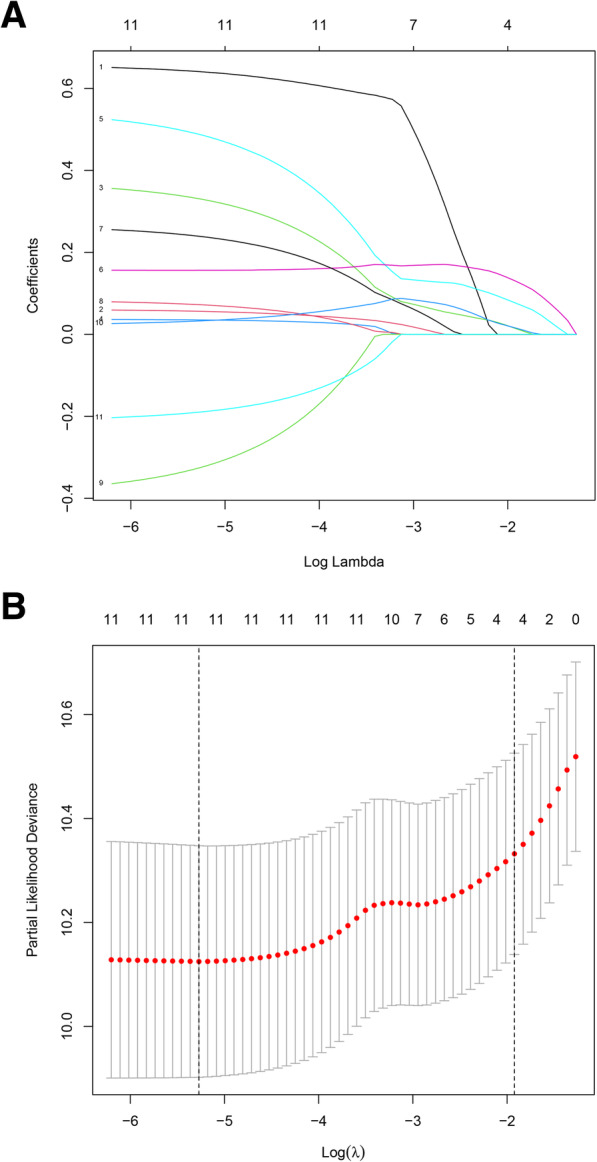
LASSO regression analysis of methylation-driven genes. **A** LASSO coefficients. **B** Plots of the ten-fold cross-validation error rates. The dotted lines indicate the minimal standard error and the optimal λ value

First, we used Kaplan–Meier (K-M) analysis to explore the connection between the gene expression of sixteen DNAm-driven genes and OS (Table S3). Eleven DNAm-driven genes were selected as candidate genes significantly related to OS (*P* < 0.05). Then, based on 1,000 repetitions of LASSO regression analyses, the 11 genes with a non-zero coefficient were selected as seed genes using 10-fold cross-validation (Fig. 4 A, B). Finally, six genes (FERMT1, LIPH, LAMA3, PPP1R14D, NQO1, VSIG2) were screened for risk score model by multivariate Cox regression. The risk score = (0.4178686 * FERMT1 mRNA level) + (0.7540802* LIPH mRNA level) + (0.2412988 * LAMA3 mRNA level) + (0.2435720 * PPP1R14D mRNA level) + (-0.4021008 * NQO1 mRNA level) + (-0.2646497 * VSIG2 mRNA level).

**Fig. 5:**
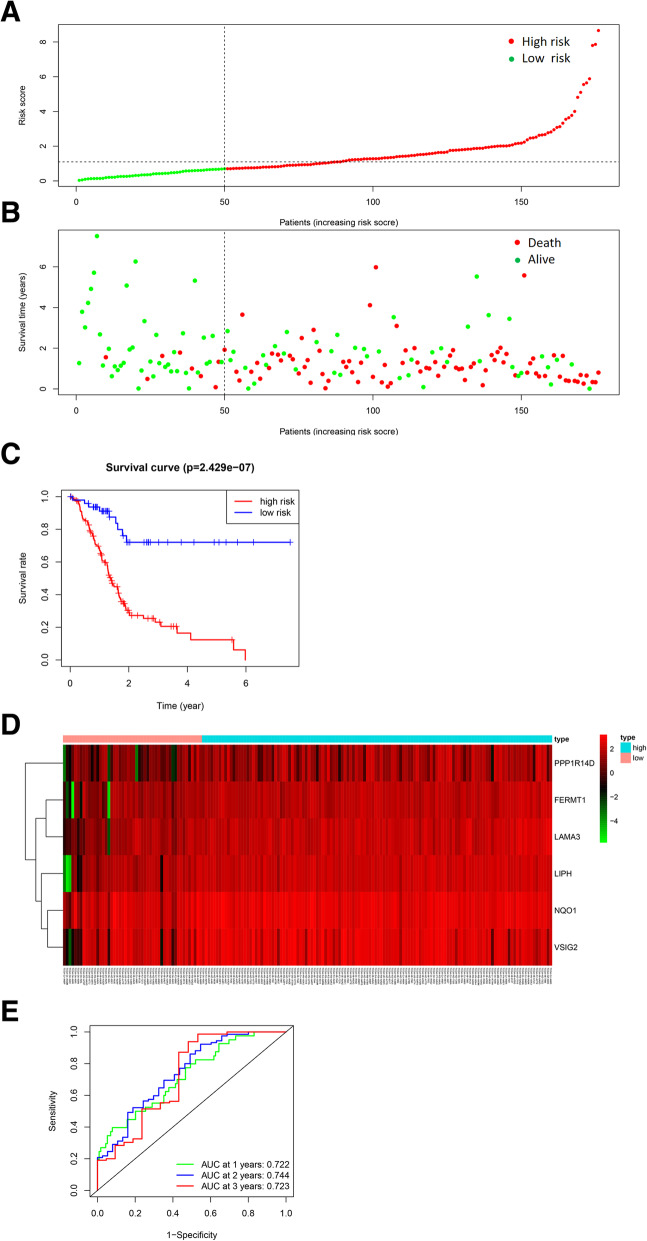
Risk score model in the TCGA database. **A** The distribution of risk score. Red dots and green dots represent the high-risk group and low-risk group, respectively. **B** Survival distribution in the high- or low- risk score group. Red dots and green dots represent deceased and live patients, respectively. **C** Kaplan–Meier survival curve of risk score. **D** Heatmap of six methylation-driven genes in the high- or low- risk score group. **E** Time-dependent ROC curve of the risk score model

We calculated the risk score of each patient and then stratified patients into low-risk and high-risk groups by X-tile. The number of deceased patients in the high-risk group was greater than in the low-risk group (Fig. 5 A, B). In the K-M analysis, the prognosis of patients in the high-risk group was statistically worse than the patients in low-risk group (*P* = 2.429e−07) (Fig. 5 C). The six-gene expression levels of patients and corresponding risk score are shown in the heatmap (Fig. 5 D). 1-year, 2year and 3-year AUC of the risk score model, which was established by the six DNAm-driven genes, was 0.722,0.744 and 0.723, respectively. (Fig. 5 E).

### External validation of the risk score model

**Fig. 6:**
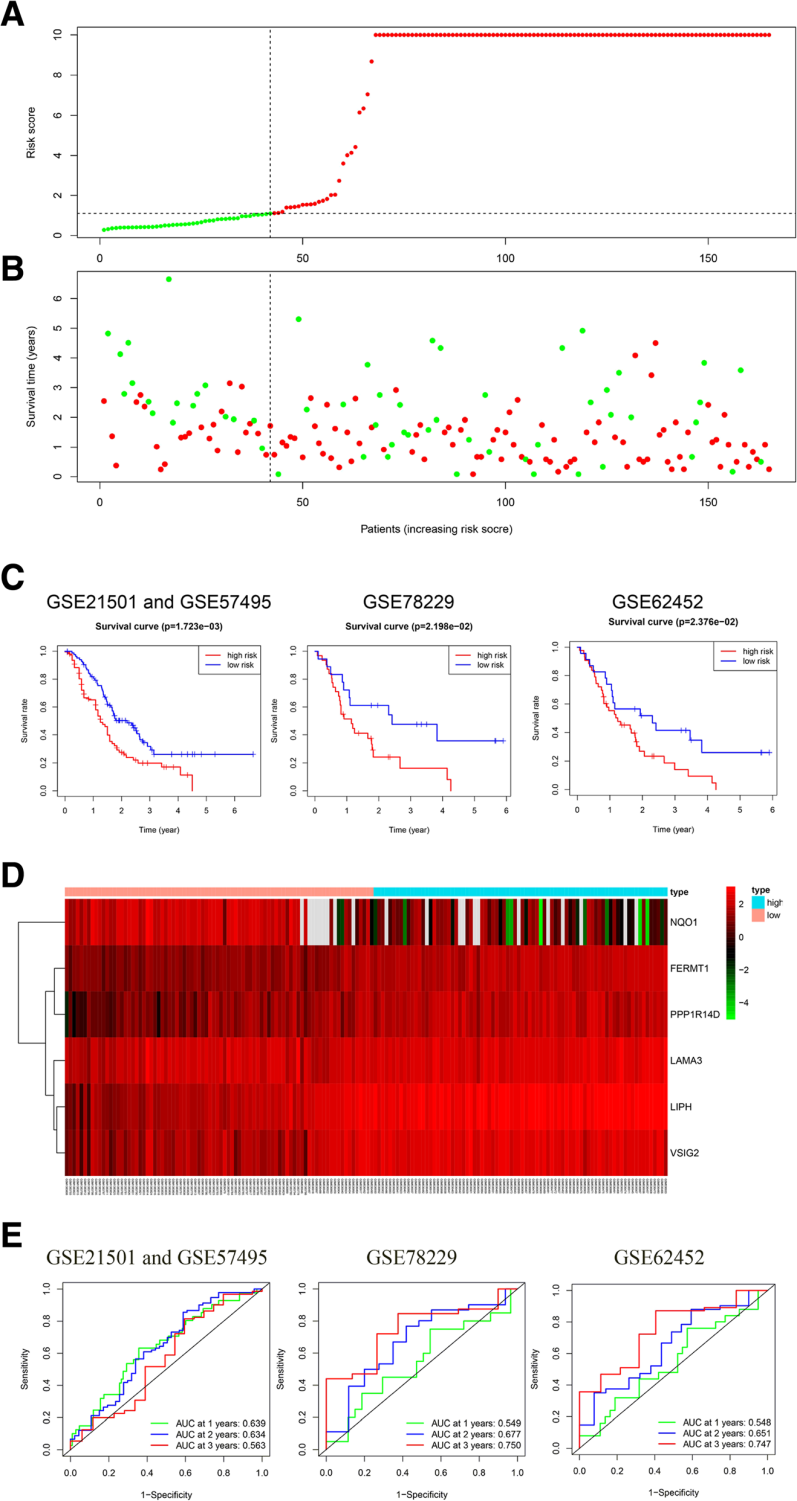
External validation of the risk score model using GEO databese. **A** The distribution of the risk score in the GSE21501 and GSE57495 merging database. Red dots and green dots represent the high-risk group and low-risk group, respectively. **B** Survival distribution in the high- or low- risk score group in the GSE21501 and GSE57495 merging database. Red dots and green dots represent deceased and live patients, respectively. **C** Kaplan–Meier survival curve of the risk score in four GEO databases. **D** Heatmap of six methylation-driven genes in the high- and low- risk subgroups in the GSE21501 and GSE57495 merging database. **E** Time-dependent ROC curve of the risk score model in four GEO databases

The validation cohorts (GSE21501, GSE57495, GSE78229 and GSE62452) were used to explore the prognostic performance of the six-gene risk score model. Risk scores of patients in the validation cohort were computed with same method as previously described. The patients were also classified into low-risk and high-risk groups by X-tile. The survival status distribution of patients in different risk groups was visualized via scatter plot (Fig. 6 A, B). The high-risk group patients had significantly worse outcomes than the low-risk group patients (*P* = 0.002, *P* = 0.022, and *P* =0.024, respectively) in the K-M survival analysis (Fig. 6 C). The distribution of the six-gene expression levels and risk scores are shown in the heatmap (Fig. 6 D). The AUC of the risk score model in the validation cohort. In the GSE78229, 1-year, 2-year and 3-year AUC of riskScore model was 0.549, 0.677 and 0.750, respectively. In the GSE62452, 1-year, 2-year and 3-year AUC of riskScore model was 0.548, 0.651 and 0.747, respectively. In the GSE21501 and GSE57495 merging databases, 1-year, 2-year and 3-year AUC of riskScore model was 0.639, 0.634 and 0.563,respectively. (Figure 6 E).

### Development and validation of the nomogram for OS prediction in PC

**Fig. 7:**
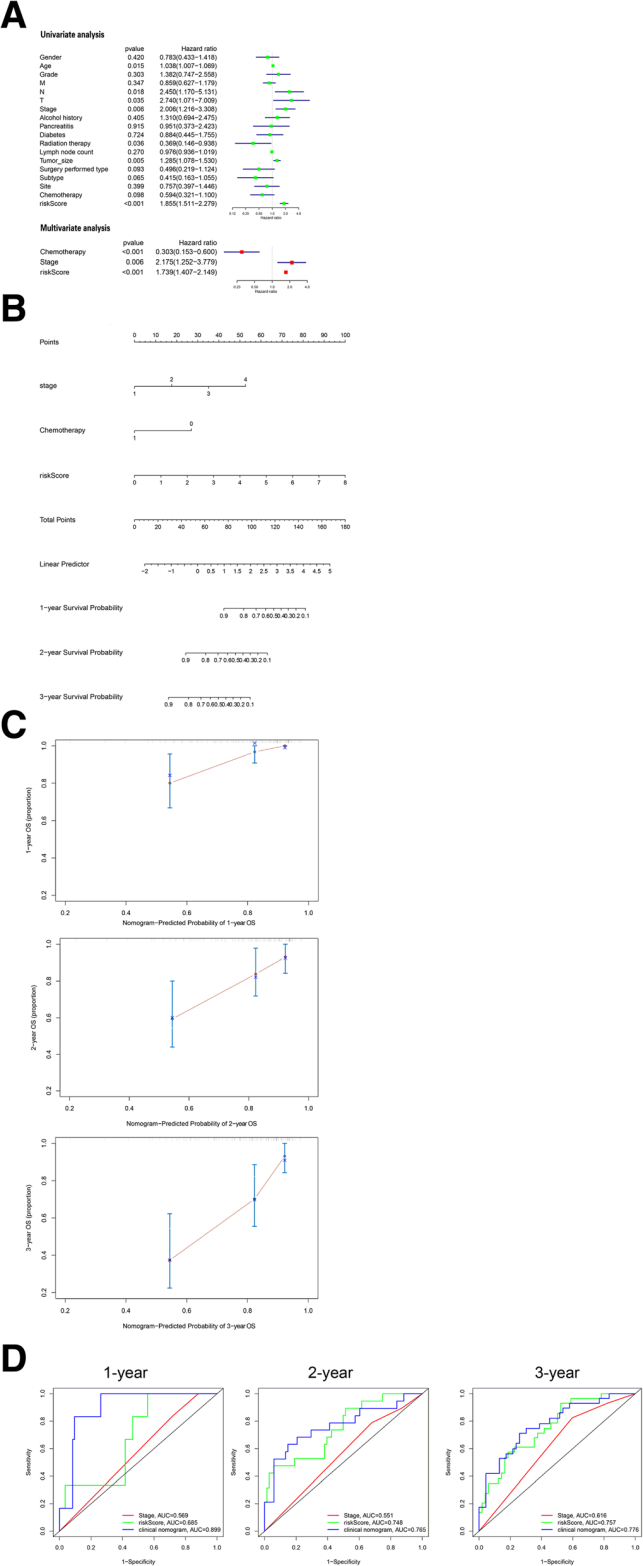
Development and validation of the nomogram in the TCGA cohort. **A** Univariate and multivariate Cox regression analysis of the risk score and clinical characteristics. **B** Nomogram to predict survival in PC patients. **C** Calibration curves of 1-, 2-, and 3-year OS. **D** ROC curves estimating the performance of the nomogram

All clinical variables were analyzed by univariate Cox regression analysis, the variables with a *P* value less than 0.1 were selected for further analysis (Fig. 7 A). Age (*p* = 0.015), T stage (*p* = 0.035; T3-4 vs. T1-2), N stage (*p* = 0.018; N1 vs. N0), AJCC TNM stage (*p* = 0.006; III-IV stage vs. I-II stage), radiation therapy history (*p* = 0.036; No vs. Yes), tumor size (*p* = 0.005), surgery type performed (*p* = 0.093; Whipple vs. Distal Pancreatectomy or others), pathological type (*p* = 0.065; Infiltrating duct carcinoma vs. other types), chemotherapy history (*p* = 0.098; No vs. Yes), and risk score (*p* < 0.001) were regarded as potential predictive variables. Finally, in the multivariate Cox regression, we used backward stepwise elimination and AIC to screen independent prognostic factors for the final nomogram model: stage, chemotherapy, and risk score (*P* < 0.05) (Fig. 7 A). We developed a nomogram model to predict 1-year, 2-year, and 3-year survival using three factors (Fig. 7B). The C-statistic of the nomogram was 0.768. The calibration of model was estimated by calibration plot using 1,000 bootstrap samples to reduce overfitting (Fig. 7 C). The 1-year, 2-year, and 3-year calibration curves presented agreement between prediction and observation. The prognostic performance of the model was also demonstrated by ROC curves. 1-year, 2-year and 3-year AUC of nomogram model was 0.899, 0.765 and 0.776, respectively (Fig. 7D).

## Discussion

In recent years, new PC cases and cancer-related deaths have been gradually increasing [2, 24]. Radical pancreatectomy for early-stage PC is a potentially curative treatment. However, PC patients are often diagnosed in the advanced stage due to a lack of typical symptoms [25]. Additionally, the efficacy of treatment in advanced PC is limited, with a median overall survival of less than 12 months [8]. Therefore, identification of effective biomarkers or the development of valid prognostic models for early diagnosis and prognosis is necessary and exigent. Most studies have combined different tumor markers with various blood parameters to improve the efficacy of diagnosis and survival prediction [26, 27]. However, levels of different blood biomarkers vary between patients and may be influenced by many factors [28]. Prognostic models established using different clinical characteristics could improve accuracy in PC [29, 30]. Nevertheless, the accuracy of these models is limited by tumor heterogeneity. Thus, it is necessary to build a new prognostic model using molecular biomarkers to further improve prognostic efficacy.

Prior studies had confirmed that tumorigenesis is correlated with aberrant methylation status, which can change expression levels of oncogenes and tumor suppressor genes [14, 31]. Numerous studies have demonstrated that DNA methylation is a specific diagnostic and prognostic biomarker [19–21]. Hence, we constructed and validated a risk score model using six DNAm-driven genes, and a nomogram prognostic model based on the risk score model and clinical predictive variables. The external validation demonstrated that the risk score model was a potential prognostic model for PC.

In our study, we identified abnormal gene methylation by comparing normal and PC samples using MethylMix. In this analysis, 16 DNAm-driven genes were screened. To explore the function of DNAm-driven genes, we performed GO analysis and pathway analysis. DNAm-driven gene function was enriched in molecular function (MF) including cell adhesion molecule binding, integrin binding, and serine−type endopeptidase activity. Function analysis and pathway analysis showed that the function of these genes could regulate tumor cell migration and metastasis.

Six genes (FERMT1, LIPH, LAMA3, PPP1R14D, NQO1, VSIG2) correlated with survival were screened by LASSO Cox regression and multivariate Cox regression. A risk model was constructed using gene expression levels and multivariate Cox regression coefficients. The survival analysis of the risk score showed that the patients with a high-risk score had worse survival status. The AUC of the risk model in the ROC curves wasgreater than 0.720. The performance of the risk model was demonstrated by external validation. To our knowledge, this is the first report of the six-gene risk model, which may be a new PC prognostic biomarker.

To further explore the prognostic value of the risk model and potential clinical variables, we developed a nomogram to calculate a score for each patient and predict survival rate. The C-index of the nomogram validated by 1,000 bootstrap resamples was 0.768. The calibration curves and ROC curves showed that the predictive ability of the nomogram was excellent.

The six genes (FERMT1, LIPH, LAMA3, PPP1R14D, NQO1, VSIG2) had high expression and hypomethylation in PC. FERMT1, named fermitin family member 1, can reduce the phosphorylation level of β-catenin and lead to the activation of the Wnt/β-catenin pathway. These changes lead to EMT (epithelial-mesenchymal transition) and have a relationship with tumor aggressiveness and invasiveness [32]. Sandra et al. demonstrated that FERMT1 was overexpressed in PC samples compared with normal samples [33]. LIPH (lipase member H), which belongs to the triglyceride lipase family, is involved in several diseases such as hypotrichosis/woolly hair, energy metabolism, and hypertensive disorder [34–36]. Although the mechanism of LIPH in tumorigenesis is unclear, numerous studies have shown that LIPH participates in tumor metastasis, and the expression level of LIPH is a predictive factor in breast cancer [37, 38]. LAMA3 (laminin subunit α3) encodes laminin, which is involved in regulating cell migration [39]. LAMA3 also regulates the expression of different types of cell growth factors that mediate cell proliferation, including KGF (keratinocyte growth factor), EGF (epidermal growth factor), and IGF (insulin-like growth factor) [40]. The LAMA3expression level in tumor tissues and its effect on survival varies between cancer types. Lin et al. found that the expression level of LAMA3 in tumor tissues was lower than in normal tissues, and the survival times of ovarian cancer patients with LAMA3 overexpression was better than those with low-expression [40]. In PC, the expression level of LAMA3 in carcinoma tissue is upregulated, and patients with high LAMA3 expression have poor outcomes [41, 42]. PPP1R14D (protein phosphatase 1 regulatory subunit 14D) is a metabolic signaling protein that is correlated with diabetes [43], and diabetes is a risk factor for PC [44]. However, the direct mechanism of PPP1R14D in tumorigenesis is still unclear. NQO1, nicotinamide adenine dinucleotide phosphate (NADPH): quinone oxidoreductase1, is a cytosolic reductase that can reduce quinones to hydroquinones using NADH or NADPH [45]. NQO1 plays an important role in protecting cells from oxidative injury via various functions [46]. Prior studies reported a relationship between abnormal expression of NQO1 and cancer [47]. Mei-Ying et al. demonstrated that NQO1 expression was higher in PC, and patients with low NQO1 expression had higher survival rates [46]. VSIG2, also called cortical thymocyte receptor, participates in antigen presentation [48]. Haimeng et al. reported that VSIG2 is a survival predictive factor in acute myeloid leukemia (AML) [49]. Nevertheless, the function of VSIG2 in PC has not yet been reported.

Our study had some limitations. First, this was a retrospective study, and the development and validation of the nomogram model was based on the TCGA dataset. Therefore, further external validation using other independent databases is necessary. Second, the sample size of PC in the TCGA database was relatively small, and only half of the patients had complete clinical information. Therefore, we also need to utilize our own data for validation.

## Conclusions

Based on the TCGA database, we screened six methylation-driven genes (FERMT1, LIPH, LAMA3, PPP1R14D, NQO1, VSIG2) associated with prognosis in PC patients. A risk score model comprising the six methylation-driven genes was established and validated to predict overall survival. The risk score was combined with different clinical factors to construct a good predictive nomogram for PC patients. Our results support the viewpoint that DNAm controlled genes are associated with prognosis. In clinical practice, use of the six DNAm-driven genes for prognosis would be a cost-effective and accurate predictive method in PC.

## Supplementary information

**Additional file 1.**

**Additional file 2.**

**Additional file 3.**

**Additional file 4.**

## Data Availability

The data used to support the findings of this study are available from TCGA (https://portal.gdc.cancer.gov/) and GEO databases (https://www.ncbi.nlm.nih. gov/geo/).
