# Supplementary figures and images for: Identification of DNA methylation-driven genes and construction of a nomogram to predict overall survival in pancreatic cancer
Source: BMC Genomics. 2021 Nov 3;22:791. doi: 10.1186/s12864-021-08097-w (PMC8567715; doi:10.1186/s12864-021-08097-w)

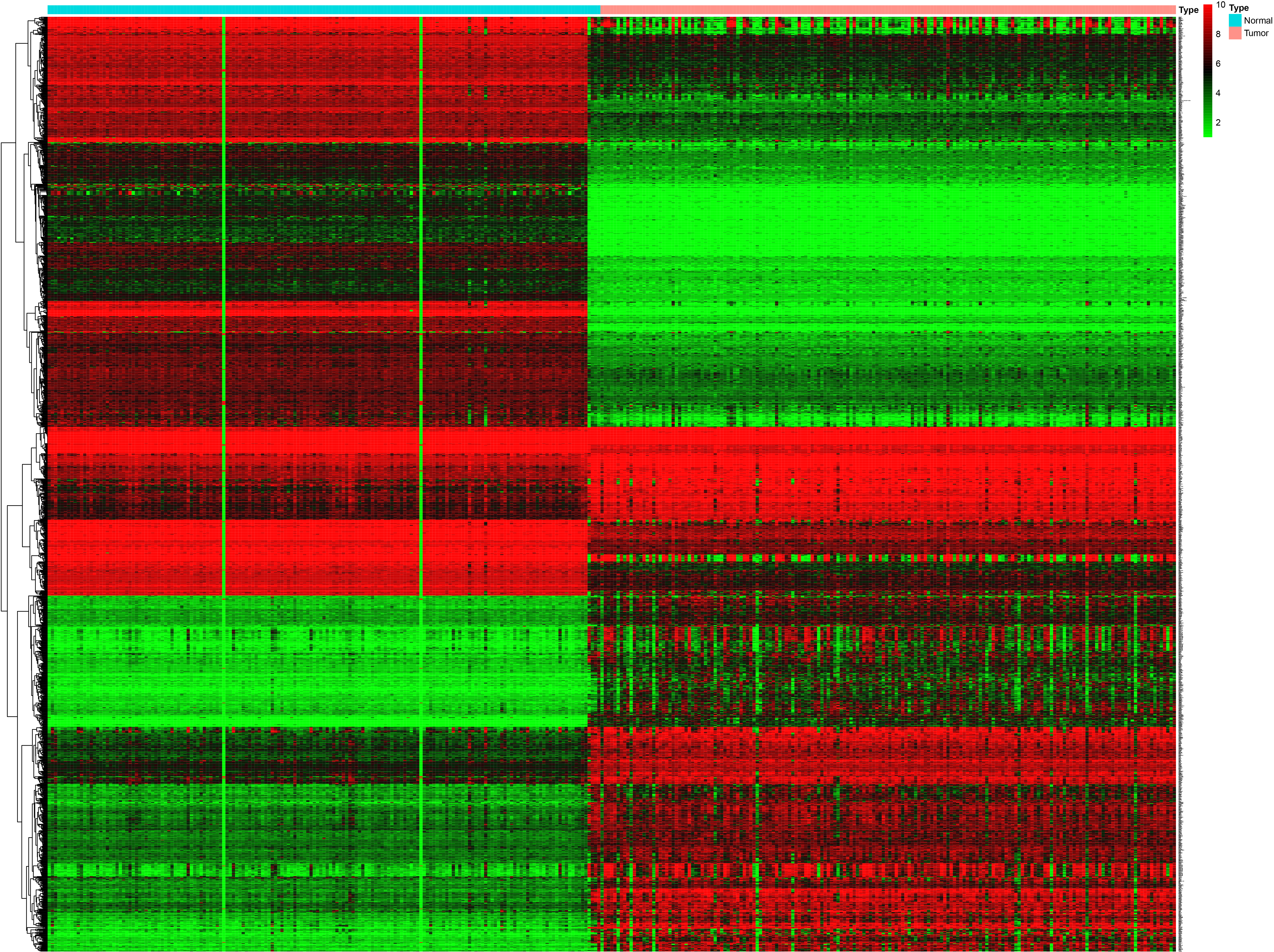


Supplementary figure1. Heatmap of 743 DEGs

Supplement: Supplementary file 1 — Additional file 1. [file 12864_2021_8097_MOESM1_ESM.doc]
